# Gastric cancer depends on aldehyde dehydrogenase 3A1 for fatty acid oxidation

**DOI:** 10.1038/s41598-019-52814-1

**Published:** 2019-11-08

**Authors:** Jae-Seon Lee, Seung Hwa Kim, Soohyun Lee, Joon Hee Kang, Seon-Hyeong Lee, Jae-Ho Cheong, Soo-Youl Kim

**Affiliations:** 1 Division of Cancer Biology, Research Institute, National Cancer Center, Goyang, 10408 Republic of Korea; 2 Department of Surgery, Yonsei University Health System, Yonsei University College of Medicine, 50 Yonsei-ro, Seodaemun-gu, Seoul 03722 Republic of Korea

**Keywords:** Drug development, Cancer metabolism

## Abstract

The major source of ATP in cancer cells remains unclear. Here, we examined energy metabolism in gastric cancer cells and found increased fatty acid oxidation and increased expression of ALDH3A1. Metabolic analysis showed that lipid peroxidation by reactive oxygen species led to spontaneous production of 4-hydroxynonenal, which was converted to fatty acids with NADH production by ALDH3A1, resulting in further fatty acid oxidation. Inhibition of ALDH3A1 by knock down using siRNA of ALDH3A1 resulted in significantly reduced ATP production by cancer cells, leading to apoptosis. Oxidative phosphorylation by mitochondria in gastric cancer cells was driven by NADH supplied via fatty acid oxidation. Therefore, blockade of ALDH3A1 together with mitochondrial complex I using gossypol and phenformin led to significant therapeutic effects in a preclinical gastric cancer model.

## Introduction

Recently, we found that cytosolic aldehyde dehydrogenase (ALDH) plays a key role in generating NADH for ATP synthesis in mitochondria^1–3^. High expression of ALDH1L1 in NSCLC catalyzes conversion of 10-formyl tetrahydrofolate (10-formyl THF) into THF, with NADH production via one carbon metabolism^1,3^. This by-produced cytosolic NADH is transported into mitochondria by the malate aspartate shuttle and used for ATP synthesis via oxidative phosphorylation (OxPhos). Knockdown of ALDH1L1 reduced ATP levels by 50%, whereas knockdown combined with inhibition of mitochondrial complex I reduced levels by 80%^1^. About 50% of NADPH is produced via the one carbon pathway^4^, which is responsible for fatty acid synthesis and regulation of reactive oxygen species (ROS). Therefore, the one carbon pathway appears to be an extra source of electron carriers such as NADH and NADPH.

However, we found that gastric cancer cells expressed high levels of ALDH3A1, which correlated inversely with overall survival among those with ALDH isotypes other than ALDH1L1. ALDH3A1 catalyzes conversion of fatty aldehydes derived from lipid peroxidation into fatty acids and NADH. The aim of this study is to investigate whether ALDH3A1 is a major contributor to ATP production via generation of NADH as an extra source of electrons in gastric cancer cells. The second aim of this study is to investigate whether pan ALDH inhibitor gossypol contributes to reduction of ATP production as well as synergistic reduction of ATP production with mitochondrial complex I inhibitor phenformin in gastric cancer cells.

## Materials and Methods

### Patients and tissue microarray analysis

A prospectively-maintained Yonsei University College of Medicine (Seoul, South Korea) Gastric Cancer cohort database was used to extract demographic and clinicopathological information and tumor tissue data obtained from 1132 patients with gastric adenocarcinoma who had undergone curative D2 gastrectomy from 2000 to 2003 at Severance Hospital. Age, sex, tumor histology, Lauren classification, and pathological TNM stage were evaluated as clinical parameters. Follow-up status was recorded and survival was calculated from the date of operation to the date of death. The median follow-up time was 112 months (range, 1–163 months). Immunohistochemical analysis of tissue microarray (TMA) containing 1161 gastric cancer tissues was performed using a Ventana XT automated stainer (Ventana Medical System, Tucson, AZ) and an anti-ALDH3A1 antibody (Abcam, Cambridge, United Kingdom). The study was waived from informed consent for study participation and approved by the institutional review board of Severance Hospital (Seoul, South Korea; 4-2015-0616). All authors confirmed that all experiments were performed in accordance with relevant guidelines and regulations.

### Immunocytochemistry

For immunofluorescence staining, cells were seeded on coverslips and treated as indicated 24 h later. After 48 h, cells were fixed for 10 min with 4% (w/v) paraformaldehyde and permeabilized for 10 min with 0.5% Triton X-100. After blocking with 3% BSA in PBS, cells were stained overnight at 4 °C with an anti-4-hydroxynonenal polyclonal antibody (ab48506; Abcam, Cambridge, United Kingdom) or an anti-ALDH3A1 antibody (ab76976; Abcam, Cambridge, United Kingdom), followed by Alexa Fluor 594-conjugated anti-mouse (A11032; Life Technologies, Carlsbad, CA, USA) or Alexa Fluor 488-conjugated anti-rabbit (A21206; Life Technologies, Carlsbad, CA, USA) secondary antibodies (diluted in 3% BSA/PBS) for 1 h at room temperature in the dark. Cells were then mounted with 4′,6-diamidino-2-phenylindole (DAPI) mounting medium to visualize nuclei (Vectashield mounting medium; Vector Laboratories, Burlingame, CA, U.S.A.). Samples were examined under a Zeiss LSM780 confocal microscope (Carl Zeiss, Oberkochen, Baden-Württemberg, Germany).

### Sulforhodamine B (SRB) cell growth assay

An SRB assay^5^ was used to measure cell proliferation as described previously^6^.

### Measurement of mitochondrial membrane potential (∆ψm)

**∆ψm** was analyzed by measuring levels of tetramethylrhodamine ester (TMRE) (87917, Sigma, St. Louis, MO, USA). Cells in 0.5 ml medium were plated in 4-well chambered cover glasses (155382, Thermo Fisher Scientific). After 24 h, cells were treated with gossypol (5 μM) and/or phenformin (100 μM). Next, 50 nM TMRE and 5 µg/ml Hoechst 33342 were added to the culture medium for 15 min at 37 °C. The 4-well chambered cover glass was placed under a LSM510 Laser Scanning Microscope. Live cell imaging was performed using Axio Observer Z1 (Carl Zeiss, Oberkochen, Germany). Relative intensity was normalized against the arithmetic mean intensity (Zen software 2.6, blue edition).

### Immunoblot analysis

Harvested cells were lysed with RIPA cell lysis buffer in the presence of a protease and phosphatase inhibitor cocktail (Sigma, St. Louis, MO, U.S.A.). The protein concentration in the cell lysates was measured using a BCA Pierce Protein Assay Kit (Thermo Fisher Scientific, Waltham, MA, U.S.A.). The same amount of protein was loaded into the wells of a 10% SDS-PAGE gel. Separated proteins were transferred to PVDF membranes, blocked with 5% BSA, and incubated overnight at 4 °C with primary antibodies (diluted in 5% BSA buffer) followed by an HRP-conjugated secondary antibody for 1 h at room temperature. Images of protein bands were captured after development with ECL reagent (Thermo Fisher Scientific, Waltham, MA, U.S.A.). The primary antibodies used for the experiments were specific for cyclin D1 (ab33929, Abcam), ALDH1L1 (ab175198, Abcam), ALDH1A1 (sc-374076, Santa Cruz Biotechnology), ALDH1L2 (ab113496, Abcam), ALDH2 (ab108306, Abcam), ALDH3A1 (ab76976, Abcam), ALDH4A1 (ab185208, Abcam), ALDH5A1 (ab65469, Abcam), and β-actin (sc-47778, Santa Cruz Biotechnology).

### Fatty acid oxidation assay

To assess the oxidation of exogenous fatty acids, the oxygen consumption rate (OCR) of cells was analyzed using a XFe96 extracellular flux analyzer (Seahorse Bioscience). Cells seeded in 60 mm dishes were transfected with NT siRNA or ALDH3A1 siRNA (40 nM). After 48 h, transfected cells were seeded in XF cell culture microplates (30,000/well) in substrate-limited medium (XF Assay Medium-Modified DMEM; Seahorse Bioscience) containing 0.5 mM glucose, 1× GlutaMAX (Gibco), 0.5 mM carnitine (Sigma-Aldrich), and 1% FBS, and then incubated for 24 h at 37 °C. To test the effect of gossypol on cellular respiration, 30,000 cells were plated in each well of a Seahorse microplate. On the next day, cells were treated for 24 h with 5 μM gossypol in substrate-limited medium. On the next day, the medium was changed to FAO assay medium (111 mM NaCl, 4.7 mM KCl, 1.25 mM CaCl_2_, 2.0 mM MgSO_4_, 1.2 mM Na_2_HPO_4_, 2.5 mM glucose, 0.5 mM carnitine, and 5 mM HEPES) for 45 min. Linoleic acid-BSA (200 µM, Sigma-Aldrich), Oleic acid-BSA (200 µM, Sigma-Aldrich) or BSA (34 µM) (Seahorse Bioscience) were added to the cells and the OCR was analyzed. Samples were mixed (3 min) and OCR was measured (3 min) in an XFe96 extracellular flux analyzer. The ATP synthase inhibitor oligomycin (3 µM), the chemical uncoupler FCCP (0.8 µM), and the electron transport inhibitor rotenone/antimycin A (2 µM) (each dissolved in DMSO) were injected at the indicated times. Oligomycin, FCCP, rotenone, and antimycin A were purchased from Sigma-Aldrich (103015-100). Raw data were normalized in an SRB assay.

### Preclinical xenograft tumor models

Balb/c-nu mice (Orientbio, Seongnam, Korea), aged between 6 and 8 weeks before tumor induction, were used for this model. This study was reviewed and approved by the Institutional Animal Care and Use Committee of the National Cancer Center Research Institute, which is an Association for Assessment and Accreditation of Laboratory Animal Care International (AAALAC International) accredited facility that abides by the Institute of Laboratory Animal Resources guide (protocols: NCC-17-397). SNU-638 cells (1.5 × 10^7^) and SK4 cells (1 × 10^7^) in 100 μL PBS were inoculated subcutaneously into mice using a 1 mL syringe. After 1 week, mice were divided into four groups: a control group treated with vehicle only, groups treated with gossypol or phenformin, and a group treated with both gossypol and phenformin. Vehicle (5% DMSO and 5% Cremophor in PBS; 100 μL) alone, gossypol (80 mg/kg/100 μL), and phenformin (100 mg/kg/100 μL) were administered orally once per day, 6 days/week, for 49 days (n = 6, mice per group). The size of the primary tumor was measured every week using calipers. Tumor volume was calculated using the formula, V u (A × B^2^)/2, where V is the volume (mm^3^), A is the long diameter, and B is the short diameter.

### Immunohistochemistry

Formaldehyde (4%)-fixed specimens were paraffin-embedded and cut at a thickness of 4 μm. Sections were dried for 1 h at 56 °C and immunohistochemical staining was performed with the automated instrument Discovery XT (Ventana medical system, Tucson Arizona, USA) using the Chromomap DAB Detection kit as follows: sections were deparaffinized and rehydrated using EZ prep (Ventana) and washed with Reaction buffer (Ventana). Antigens were retrieved by heating at 90 °C for 30 min in Citrate buffer (pH 6.0; Ribo CC, Ventana) prior to detection with an anti-Ki-67 antibody (ab15580; Abcam, Cambridge, UK).

### Statistical analysis

Statistical analysis was performed using the Student’s t test as appropriate. Tumor growth was analyzed by two-way analysis of variance tests. All analyses were performed using GraphPad PRISM 5 (GraphPad Software, San Diego, CA, USA).

### Supplementary materials and methods

Detail methods of Cell culture, XF Cell Mito Stress analysis, Relative quantitation of metabolites using liquid chromatography-tandem mass spectrometry (LC-MS/MS), Relative quantitation of free fatty acids using gas chromatography-mass spectrometry (GC-MS), Relative quantitation of fatty acyl CoA using LC-MS/MS, Cell cycle analysis, TUNEL assay: cell death detection are provided in the Supplementary Materials and methods.

## Results

### Gastric cancer tissues and cell lines show high expression of ALDH3A1

**Figure 1:**
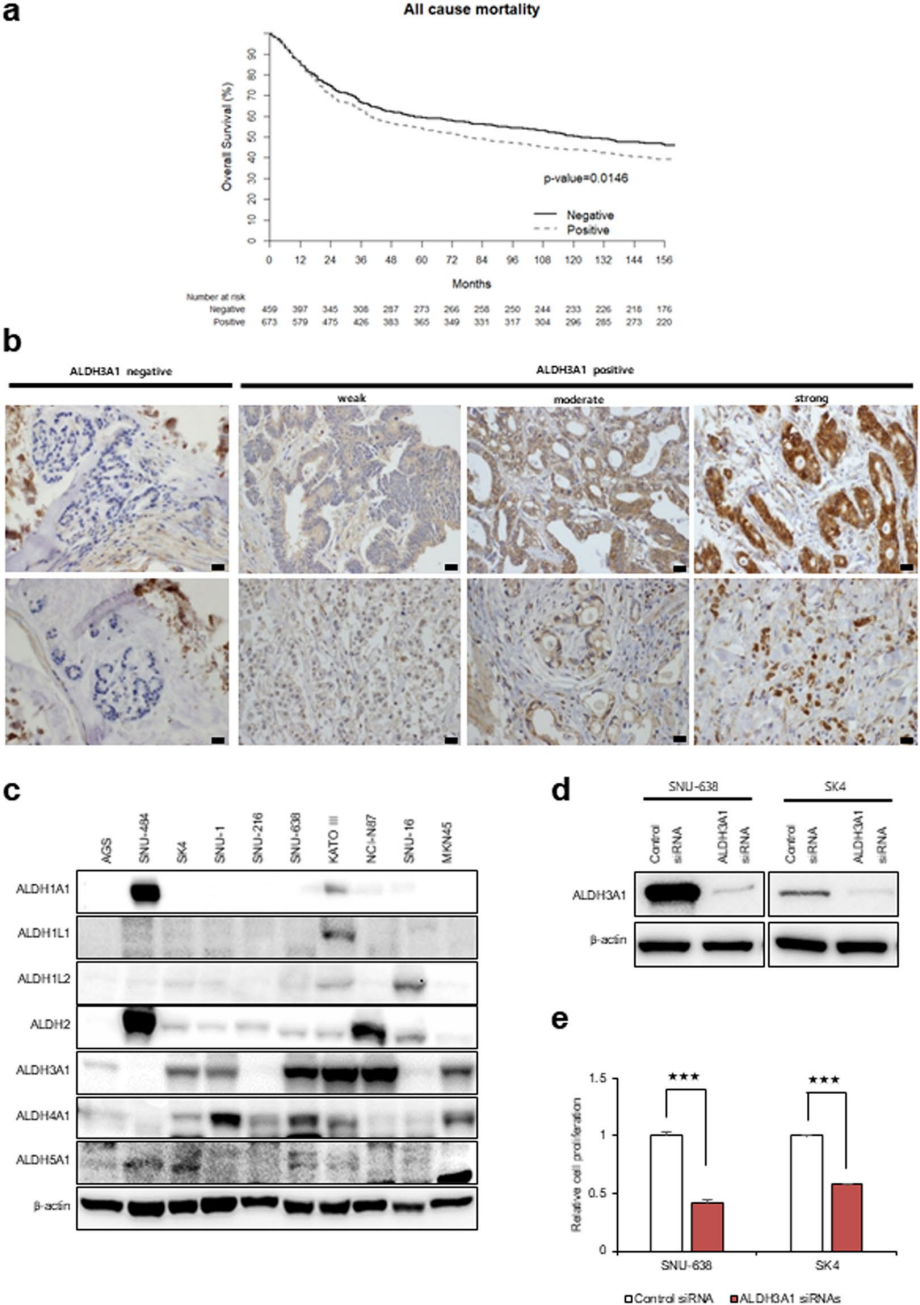
Expression of ALDH3A1 is associated with poor survival of patients with gastric cancer. Knockdown of ALDH3A1 inhibits proliferation of gastric cancer cells. (**a**) Kaplan–Meier survival curve. GC patients positive for ALDH3A1 (n = 673) had a worse prognosis than those that were negative for ALDH3A1 (n = 459) (p = 0.0146, log-rank test). (**b**) Representative images of immunohistochemical staining of ALDH3A1. Scale bar Re20 µm. (**c**) Western blot showing higher expression of ALDH3A1 by gastric cancer cell lines compared with other ALDH isotypes. (**d**) Small interfering (si)RNA-mediated knockdown of ALDH3A1, as assessed by Western blotting. (**e**) Knockdown of ALDH3A1 suppressed proliferation of gastric cancer cells in an SRB assay. Data are expressed as the mean and standard deviation of three independent experiments. *p <0.05, **p <0.01, ***p <0.001.

To investigate the clinical significance of ALDH3A1 in gastric cancer, we analyzed expression of ALDH3A1 protein along with clinical outcomes using 1132 gastric cancer tissue samples in a TMA. Increased expression of ALDH3A1 protein showed a significant association with a poor prognosis (p = 0.016, log-rank test; Fig. 1a,b). Next, we investigated whether expression of ALDH3A1 protein in 1132 TMA samples correlated with clinicopathological parameters. Although there was no difference in sex and TNM stage between the ALDH3A1 high and low expression subgroups, there was a significant different in tumor histology (Table S1). Among the isotypes of ALDH, stomach cancer cells showed increased expression of ALDH3A1 (Fig. 1c). To test whether expression of ALDH3A1 affects proliferation of stomach cancer cells, ALDH3A1-expressing SNU638 and SK4 cells were transfected with siRNA targeting ALDH3A1 and cell growth was measured (Fig. 1d,e). Knockdown of ALDH3A1 in gastric cancer cells inhibited cell growth by about 50–70% over 48 h.

### Knockdown or inhibition of ALDH3A1 reduces fatty acid oxidation and ATP production

**Figure 2:**
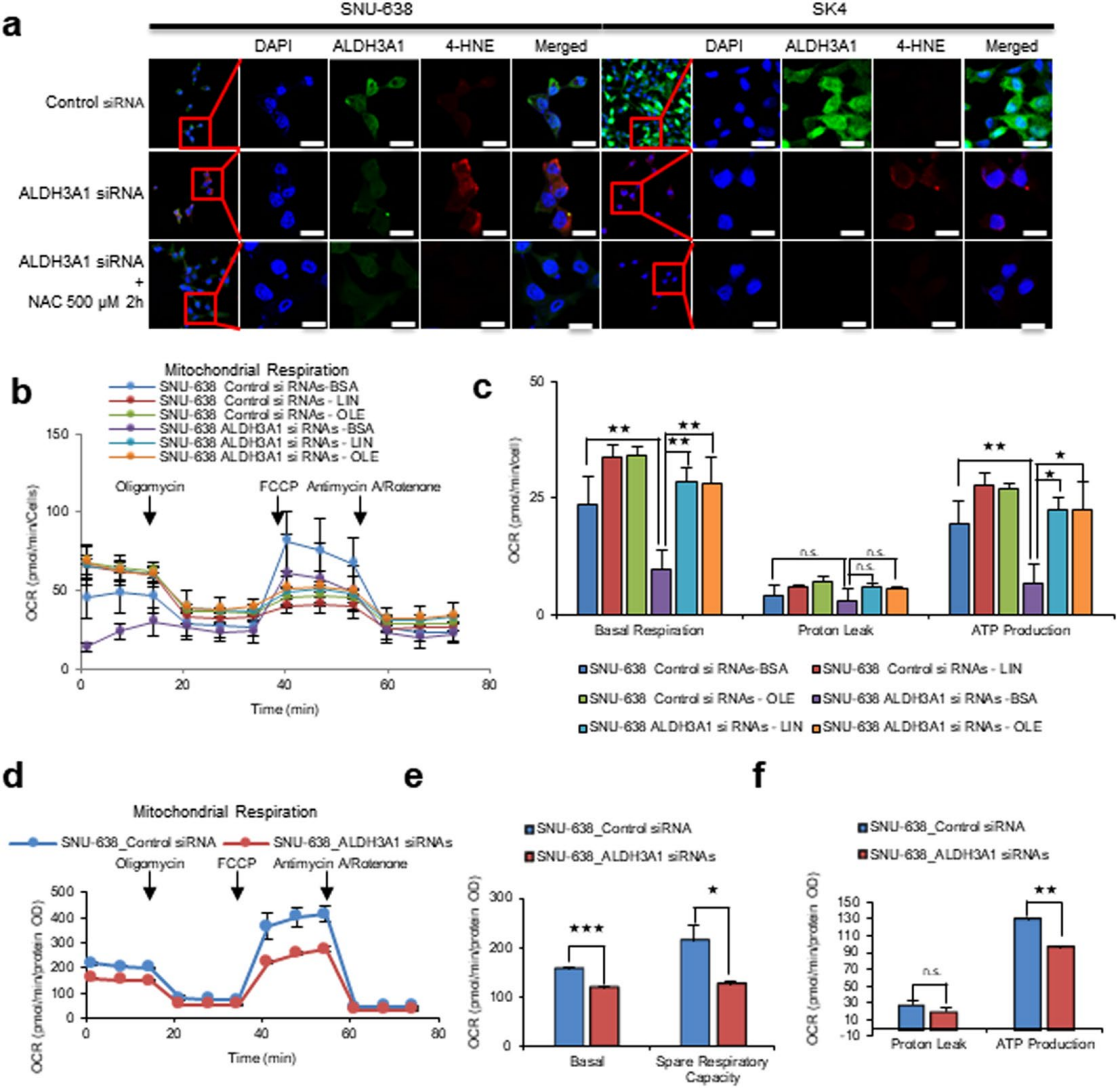
Knockdown of ALDH3A1 induces production of 4-HNE and decreases β-oxidation in gastric cancer cells. (**a**) Knockdown of ALDH3A1 increases levels of 4-hydroxynonenal in SNU-638 and SK4 gastric cancer cells (as measured by immunocytochemical analysis). Scale bar  S20 μm. (**b**,**c**) Seahorse XF analysis of SNU-638 cells treated sequentially with oligomycin, the chemical uncoupler FCCP, and antimycin A/Rotenone (downward arrows) in the presence of linoleic acid-BSA, oleic acid-BSA or BSA. Fatty acid oxidation in ALDH3A1-knockdown cells was lower than that in control cells. (**d**–**f**) ALDH3A1 knockdown in SNU-638 cells reduced oxygen consumption rates and respiratory parameters and oxygen consumption rates were measured with non-lipid substrate. Abbreviations: 4-hydroxynonenal (4-HNE); 4′,6-diamidino-2-phenylindole (DAPI); N-Acetylcysteine (NAC); Carbonyl cyanide-4-(trifluoromethoxy)phenylhydrazone (FCCP); Linoleic acid (LIN); Oleic acid (OLE). Data are expressed as the mean and standard deviation of three independent experiments. *p <0.05, **p <0.01, ***p <0.001.

In cancer cells, fatty acids such as linoleic acid and arachidonic acid must be peroxidized by ROS and converted to fatty aldehydes^7^. To test whether ALDH3A1 plays an important role in fatty acid oxidation, we performed immunocytochemical staining of 4-hydroxynonenal (4-HNE) after ALDH3A1 knockdown (Fig. 2a). The results revealed an inverse correlation between HNE staining and ALDH3A1 expression in SNU-638 and SK4 cells. This implies that ROS-mediated fatty acid peroxidation occurs spontaneously in cancer cells, resulting in production of HNE. Treatment with a ROS inhibitor, NAC, together with ALDH3A1 knockdown abrogated accumulation of 4-HNE in SNU-638 and SK4 cells (Fig. 2a). This suggests that fatty aldehydes are produced due to spontaneous peroxidation by ROS as an electron donor, which leads to generation of hydroxynonenoic acid (HNA) by ALDH3A1 for further downstream fatty acid oxidation (β-oxidation) in gastric cancer cells.

To test whether decreased expression of ALDH3A1 affects fatty acid oxidation, we measured β -oxidation by treating gastric cancer cells (in which ALDH3A1 was knocked down) with linoleic acid or oleic acid under fatty acid-restricted conditions (Fig. 2b). SNU-638 cells were incubated for 24 h in substrate-limited medium (0.5 mM glucose, 0.5 mM carnitine, and 1% FBS). Next, ALDH3A1 was knocked down and cells were incubated in substrate-limited medium for another 24 h, and the medium was changed to FAO assay medium (2.5 mM glucose, 0.5 mM carnitine) for 45 min. Linoleic acid-BSA (200 µM), Oleic acid-BSA or BSA was added, and the OCR was measured. After linoleic acid or oleic acid treatment, SNU-638 cells showed a ~40% increase in OCR and ATP production (Fig. 2b,c). After knockdown of ALDH3A1, basal β -oxidation and ATP production by SNU-638 fell by ~60% compared with that in the control (Fig. 2c). Fatty acid oxidation generates electron sources, such as NADH and FADH_2_, which are converted to ATP through electron transfer complex (ETC) I–V; this is an oxygen-consuming process called OxPhos. To test whether ALDH3A1 expression is related to OxPhos, we measured the OCR after siRNA-mediated knockdown of ALDH3A1 (Fig. 2d). The OCR of SNU-638 with inactive ALDH3A1 fell by ~40% (spare respiratory capacity) and ATP production fell by about 25% (Fig. 2d–f).

**Figure 3:**
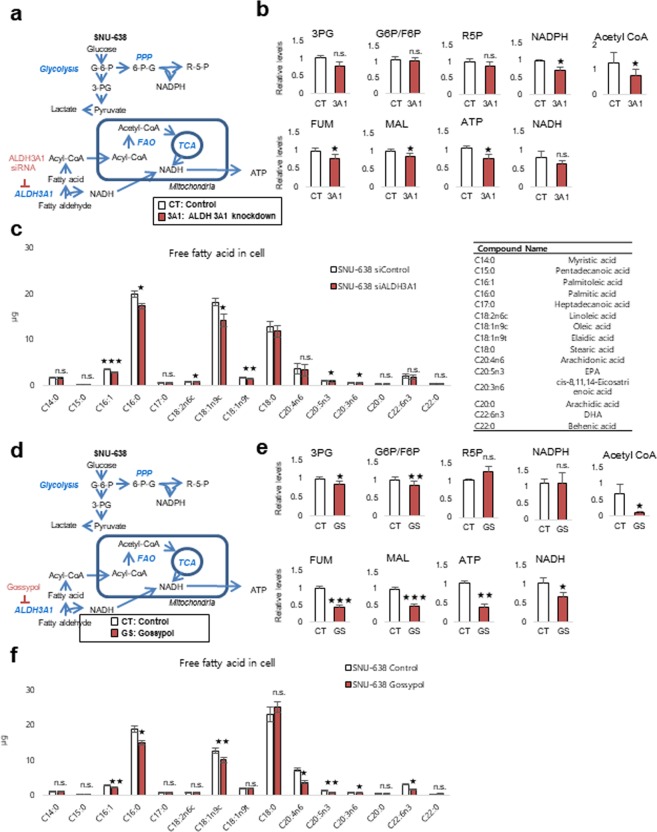
Knockdown of ALDH3A1 leads to a marked reduction in production of NADH and ATP by SNU-638 cells, along with a concomitant fall in acetyl-CoA levels. (**a**–**c**) Effect of ALDH3A1 siRNA treatment (40 nM for 48 h) on metabolites derived from various metabolic pathways in SNU-638 cells. (**d**–**f**) Effect of gossypol treatment (5 µM) on metabolites derived from various metabolic pathways in SNU-638 cells. Relative pool sizes of metabolites after ALDH3A1 siRNA treatment for 48 h or gossypol treatment for 24 h were assessed by targeted LC-MS/MS and GC-MS. Abbreviations: 3-phosphoglycerate (3PG); Glucose 6-phosphate (G6P); Fructose 6-phosphate (F6P); Ribose 5-phosphate (R5P); Fumarate (FUM); Malate (MAL). Data are expressed as the mean and standard deviation of three independent experiments. *p <0.05, **p <0.01, ***p <0.001.

To test whether ATP production depends on fatty acid oxidation through ALDH3A1, metabolite analysis was performed in cells subjected to ALDH3A1 knockdown for 24 h (Fig. 3a). ALDH3A1 knockdown in SNU-638 cells had no significant effect on metabolites derived from the glycolysis and pentose phosphate pathways, whereas metabolites derived from the TCA cycle (intermediates such as fumarate and malate) fell by approximately 20% when compared with levels in the untreated group (Fig. 3b). ATP production fell by approximately 40% after knockdown of ALDH3A1 (Fig. 3b), which was accompanied by a fall in levels of mid-chain fatty acids, including palmitic acid, oleic acid, and eicosapentaenoic acid (EPA), along with a ~40% decrease in acetyl-CoA (Fig. 3c). We also performed metabolite analysis in cells treated for 24 h with gossypol and inhibitor of ALDH (Fig. 3d). Gossypol acts as a reversible noncompetitive inhibitor of ALDHs, although it is more selective for the ALDH3 isozyme than for the ALDH1 and ALDH2 isozymes^8^. Gossypol may interact with the cofactor binding site. ALDH inhibition had no significant effect on glycolysis and pentose phosphate pathway metabolites, including NADPH; however, it reduced the amounts of TCA cycle intermediates by approximately 60% compared with those in untreated cells (Fig. 3e). ATP production fell by 60% (Fig. 3e) and was accompanied by a reduction in the amounts of mid-chain fatty acids, including palmitic acid, oleic acid, and EPA, along with a 90% reduction in acetyl-CoA (Fig. 3f). This suggests that gastric cancer cells depend on fatty acids to generate acetyl-CoA for use in the TCA cycle through fatty aldehydes.

**Figure 4:**
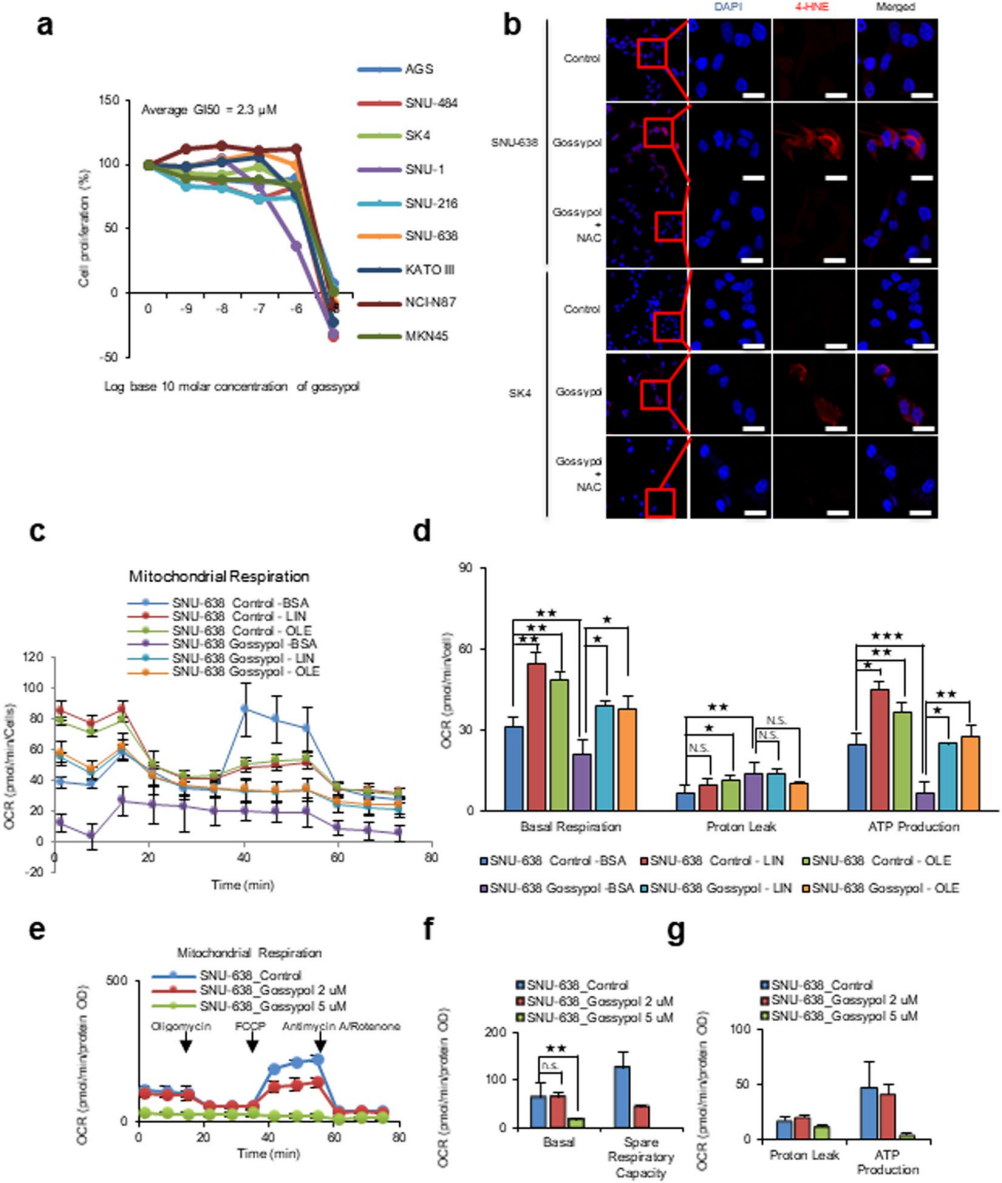
Gossypol increases 4-HNE levels and decreases β-oxidation in gastric cancer cells. (**a**) Gossypol (48 h) inhibits proliferation of various gastric cancer cell lines, as determined in a SRB assay. (**b**) Immunocytochemistry analysis shows that treatment with 5 μM gossypol for 48 h increases 4-hydroxynonenal levels in SNU-638 and SK4 cells. Treatment of cells with 500 μM NAC for 2 h after gossypol treatment ameliorates the effects of gossypol on 4-HNE. Scale bar of20 μm. (**c**,**d**) Seahorse XF analysis of SNU-638 cells treated with 5 μM gossypol for 24 h, followed by sequential treatment with oligomycin, the chemical uncoupler FCCP, and antimycin A/Rotenone (downward arrows) in the presence of linoleic acid-BSA, oleic acid-BSA or BSA. Fatty acid oxidation in SNU-638 cells treated with gossypol was lower than that in control cells. (**e**–**g**) Gossypol treatment of SNU-638 cells for 24 h reduced the oxygen consumption rate and other respiration parameters and oxygen consumption rates were measured with non-lipid substrate. Data are expressed as the mean and standard deviation of three independent experiments.

To test whether ALDH inhibitors have the same effect on cell proliferation as ALDH knockdown, various gastric cancer cells were exposed to the pan-ALDH inhibitor gossypol^9^ (Fig. 4a). Gossypol inhibited cancer cell proliferation with a GI50 (The concentration for 50% of maximal inhibition of cell proliferation) of 2.3 µM (Fig. 4a). Gossypol treatment of SNU-638 and SK4 cells caused an increase in 4-HNE (Fig. 4b) (as did siRNA-mediated inhibition of ALDH3A1; Fig. 2a); this was reversed by co-treatment with the anti-oxidant NAC (Fig. 4b). To test whether gossypol-mediated inhibition of ALDH affects fatty acid oxidation, we measured β-oxidation in gastric cancer cells treated with linoleic acid or oleic acid under fatty acid-restricted conditions (as in Fig. 2b). The effects of gossypol on fatty acid oxidation were analyzed by measuring the OCR (Fig. 4c,d). The OCR of linoleic acid-BSA or oleic acid-BSA-treated SNU-638 cells increased by about ~70% when compared with that in untreated SNU-638 cells. Gossypol reduced the basal capacity of OCR in linoleic acid or oleic acid-treated cells by about 30% (Fig. 4d) and ATP levels in linoleic acid or oleic acid-treated cells fell by 40% (Fig. 4d). Next, we measured the effect of gossypol on total OCR in SNU-638 cells (Fig. 4e). Cells were grown under normal conditions (high glucose and 10% FBS) and then treated for 24 h with 5 µM gossypol; basal OCR fell to 10% of that in control cells, accompanied by a 90% decrease in ATP production (Fig. 4f,g).

### Combination treatment with gossypol and phenformin induces significant cancer cell death following ATP depletion

**Figure 5:**
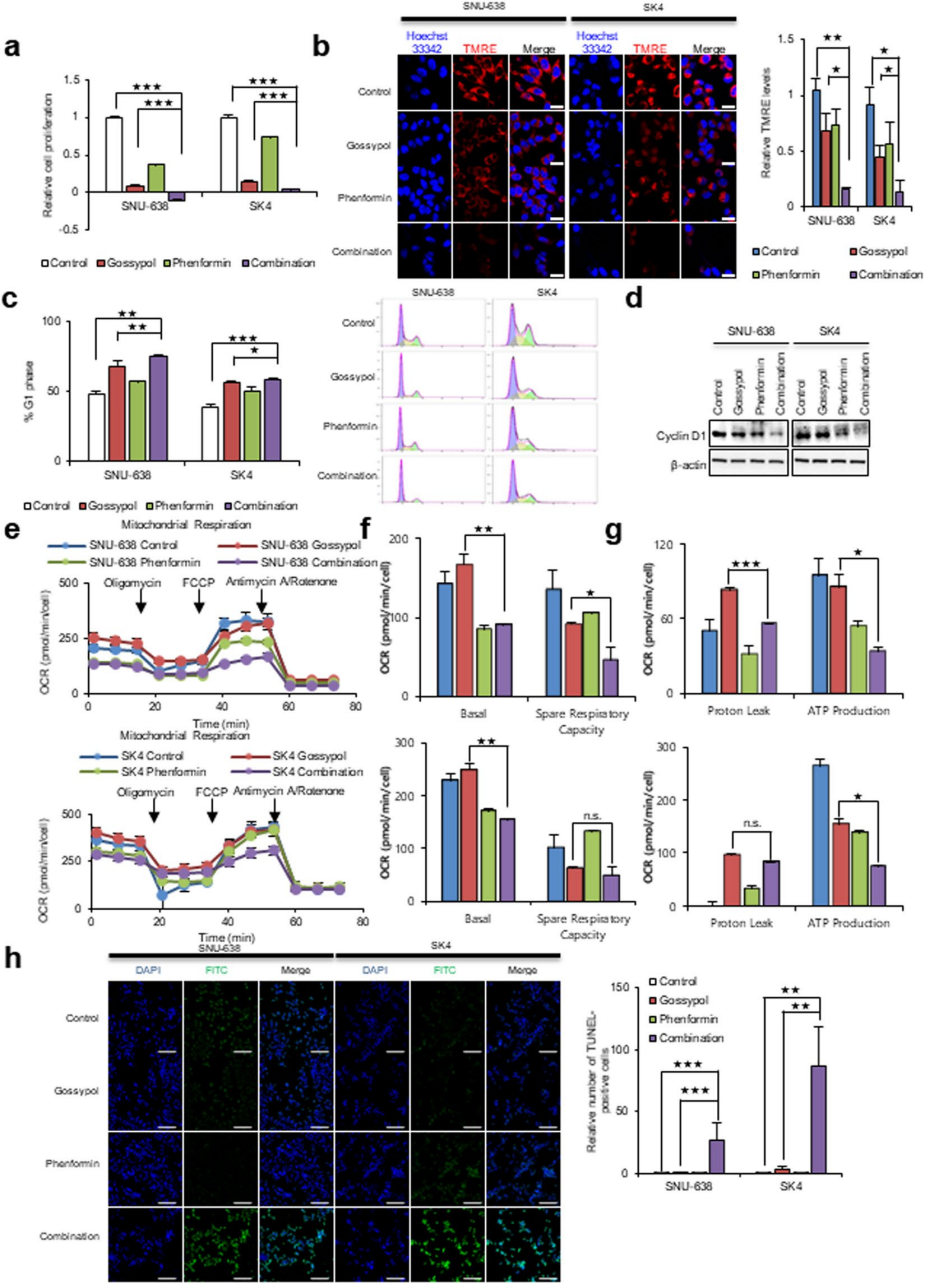
Treatment with gossypol plus phenformin led to a synergistic reduction in cell proliferation via decreased ATP production. (**a**) Combined treatment of SNU-638 and SK4 cells with 5 μM gossypol and 100 μM phenformin for 48 h led to synergistic inhibition of cell growth. (**b**) Combined treatment for 24 h reduced the mitochondrial membrane potential in SNU-638 and SK4 cells, as determined by TMRE staining and live cell imaging. Scale bar = 20 μm. (**c**) Combined treatment led to a synergistic increase in cell cycle arrest of SNU-638 and SK4 cells at G1/S transition after 24 h, as analyzed by flow cytometry. (**d**) Combined treatment also reduced expression of cyclin D1 after 24 h, as analyzed by western blotting. (**e**–**g**) Combined treatment of SNU-638 and SK4 cells with 5 μM gossypol and 10 μM phenformin (12 h) led to a synergistic reduction in oxygen consumption rates and respiration parameters. (**h**) Treatment of SNU-638 and SK4 cells with 5 μM gossypol plus 100 μM phenformin (24 h) led to a synergistic increase in cell death (determined in a TUNEL assay). Scale bar = 200 μm. Abbreviations: Tetramethylrhodamine, ethyl ester (TMRE). Data are expressed as the mean and standard deviation of three independent experiments. *p <0.05, **p <0.01, ***p <0.001.

Fatty acid-derived acetyl-CoA is catabolized to CO_2_ and NADH via the TCA cycle. Finally, ATP is produced from NADH via OxPhos. Therefore, blocking OxPhos in cancer cells using the complex I inhibitor phenformin may increase ATP depletion and inhibit ALDH. To increase the inhibitory effect of OxPhos, cells were co-treated with gossypol and phenformin (Fig. 5). Combined treatment of SNU-638 and SK4 cells with gossypol and phenformin had a synergistic anti-proliferative effect (Fig. 5a). Under these conditions, the **∆ψm** was reduced by about 85% (Fig. 5b). Reduction in mitochondrial activity affects cell cycle efficiency in cancer cells. Cell cycle analysis of cells treated for 24 h with gossypol and phenformin revealed a 50% increase in the number of cells in G1 phase (Fig. 5c). This implies that ATP depletion induces G1 arrest; this was confirmed by immunoblot analysis of cyclin D1 expression, which showed reduced levels in treated SNU-638 and SK4 cells (Fig. 5d). To test the effect of gossypol/phenformin treatment on mitochondrial respiration, we examined the OCR after SNU-638 and SK4 cells were treated with gossypol, phenformin, or a combination of the two for 12 h (Fig. 5e). We found that treatment with 5 µM gossypol (Fig. 5e) caused no change in basal respiratory capacity; this is a stark contrast to the results in Fig. 4d, which show a 74% decrease in basal respiratory capacity. The reason for this is that the cells in Fig. 4d were subjected to combined treatment for 24 h to show the effect of synergy, whereas those in Fig. 5e were treated with a single agent for 12 h. We also found that the OCR spare respiratory capacity of SNU-638 cells was reduced by 34%, 24%, and 67% after exposure to gossypol, phenformin, and combination treatment, respectively, and that the OCR spare respiratory capacity of SK4 cells was reduced by 38%, 0%, and 52% after exposure to gossypol, phenformin, and combination treatment, respectively. (Fig. 5f). ATP production by SUN-638 cells fell by 10%, 50%, and 70%, respectively, and ATP production by SK4 fell by 42%, 48%, and 73% after exposure to gossypol, phenformin, and combination treatment, respectively (Fig. 5g). These results imply that phenformin is an effective regulator of basal respiration capacity, while gossypol is an effective regulator of spare respiratory capacity; this results in effective synergistic downregulation of overall OCR (Fig. 5e–g). Combination treatment with gossypol and phenformin induced significant cell death (Fig. 5h). A TUNEL assay showed that death of SNU-638 and SK4 cells increased by about 30- and 80-fold, respectively (Fig. 5h). This result concurs with previous results showing that combined treatment of NSCLC for 24 h with gossypol and phenformin induced cell death following cell cycle arrest^1^.

### Gossypol/phenformin shows significant anti-cancer effects in a preclinical model of gastric cancer

**Figure 6:**
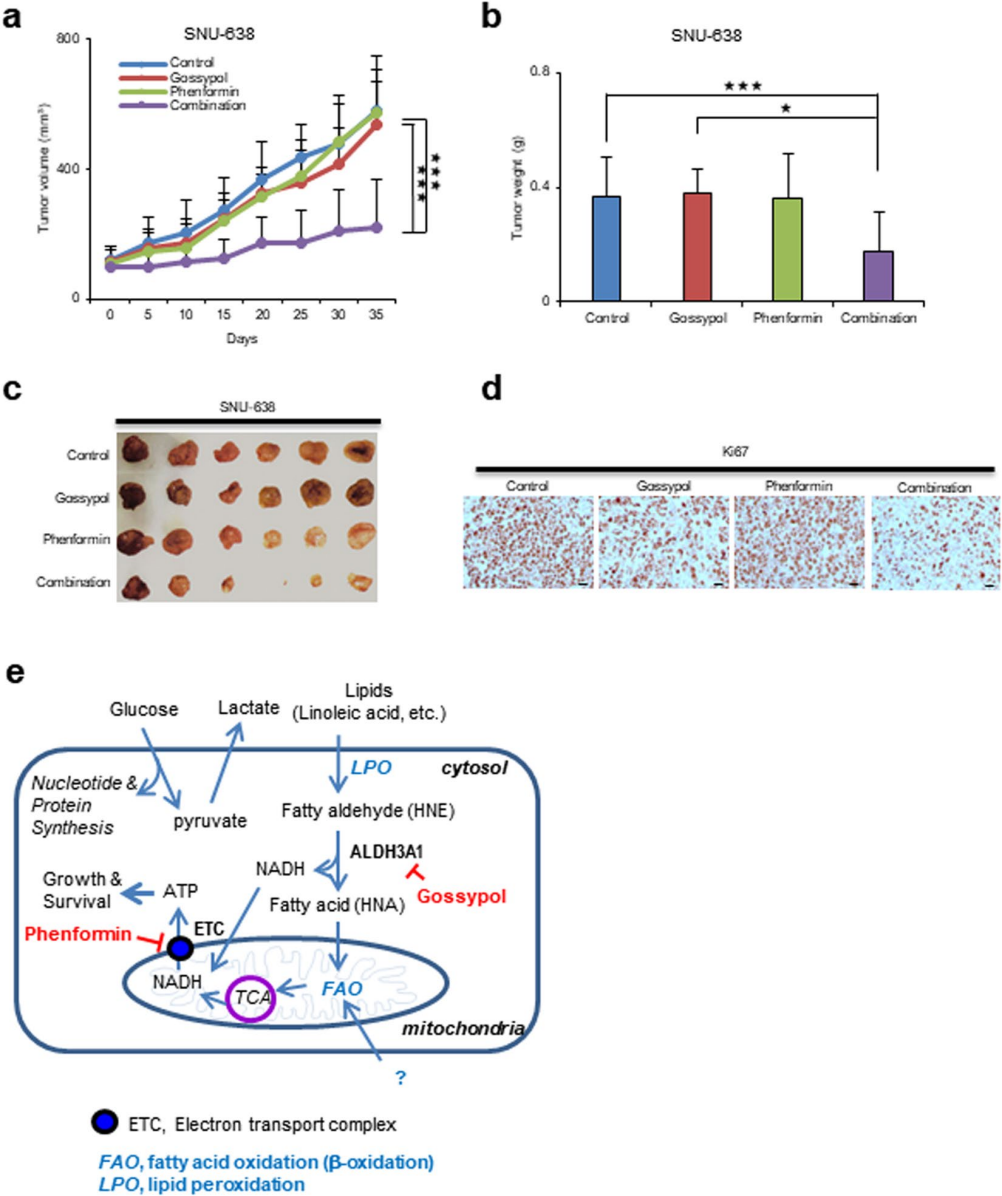
Combined treatment with gossypol plus phenformin led to synergistic suppression of tumor growth in a mouse model of human gastric cancer. (**a**) SNU-638 (1.5 × 10^7^) cells were injected into BALB/c nude mice (6–8 weeks old). When the volume of the tumor mass reached 110 mm^3^, mice were assigned randomly to one of four treatment groups (n = 6 per group): vehicle control, gossypol, phenformin, and gossypol plus phenformin. Gossypol (80 mg/kg body weight), phenformin (100 mg/kg body weight), and vehicle were administered orally 6 days/week. The graph shows a synergistic reduction in tumor growth after combined treatment with gossypol and phenformin. (**b**) The final weight of subcutaneous tumors derived from SNU-638 cells. (**c**) Representative photograph of subcutaneous tumors derived from SNU-638 cells. (**d**) IHC analysis of Ki67 staining in SNU-638 tumor xenograft tissues. Scale bar IH20 µm. (**e**) A proposed model for the synergistic mechanism underlying inhibition of ALDH3A1 and mitochondria complex 1 in gastric cancer. *p <0.05, **p <0.01, ***p <0.001.

We found that combined treatment of various gastric cancer cell lines with gossypol and phenformin induced cell death *in vitro* (Fig. S1). Therefore, we asked whether combined inhibition of ALDH and OxPhos had synergistic therapeutic effects in a mouse model of gastric cancer (Figs 6 and S3). Cultured SNU-638 (Fig. 6) and SK4 (Fig. S3) cells were injected subcutaneously (near the scapulae) into 6–8-week-old female nude BALB/c mice. Oral administration of gossypol (80 mg/kg), phenformin (100 mg/kg), and gossypol (80 mg/kg) plus phenformin (100 mg/kg) was initiated when tumors reached a volume of 100 mm^3^; dosing continued for 6 days per week over 5 weeks. The body weight of treated and control mice did not differ over the course of the experiment (Figs S2 and S3c). Combination treatment reduced the weight of SNU-638 and SK4 tumors to 50% and 60% that of controls, respectively; by contrast, single administration of gossypol or phenformin had no therapeutic effect (Figs 6a,b; S3a,b). After 5 weeks, tumor volumes in mice receiving combined treatment were significantly lower than those in vehicle-treated controls and mice receiving single agents (Fig. 6c). Immunohistochemical analysis revealed that expression of Ki67 was significantly lower in the combined treatment group (Fig. 6d).

## Discussion

Studies identified many candidate biomarkers for gastric cancer, such as choline, lactate, propanol, fumarate, and fatty acids^10–12^. Some predictive models were developed using metabolite algorithms based on 18 metabolites, including 1-acyl-lysophosphatidylcholine and poly unsaturated fatty acids, resulting in 90% accuracy for predicting chemosensitivity of gastric cancer^11^. Recently, reviews of common metabolic targets for cancer therapy suggested targeting mostly metabolic biosynthesis pathways that drive cancer proliferation; these include glycolysis, the one carbon pathway, TCA cycle intermediates, serine biosynthesis, and fatty acid synthesis^13–15^. However, no single specific therapeutic metabolic target has been identified. In the present study, we found that cancer-specific metabolic characteristics include spontaneous peroxidation of fatty acids (such as linoleic acid and arachidonic acid) by ROS, which supplies the fatty aldehyde HNE as an electron donor; this is then converted to fatty acid HNA and NADH by ALDH3A1. HNA and NADH are transported to the mitochondria through CPT and MAS, respectively, for use in fatty acid oxidation and electron transport. Furthermore, metabolic profile analysis revealed that most acetyl-CoA used in the TCA cycle is derived from fatty acid oxidation (β-oxidation) in the mitochondria. We showed that blocking ALDH3A1 and mitochondrial complex I using gossypol and phenformin, respectively, had synergistic anti-cancer effects in a preclinical gastric cancer model. ALDH3A1-mediated production of NADH from fatty aldehydes is a quite unique process that provides extra electrons for ATP production; this is because HNE is a harmful byproduct generated by peroxidation. Indeed, HNE is produced when n-6-polyunsaturated fatty acids such as linoleic acid are attacked by ROS^16^.

It is suggested that fatty acids may be the major source of energy metabolism in cancer cells through β-oxidation in the mitochondria^17,18^. Increased expression of fatty acid receptor CD36 is associated with metastasis of human oral carcinoma^19^. Blocking fatty acid transporter CD36 impairs metastasis of human melanoma- and breast cancer-derived tumors^19^. Lipid metabolites, including free fatty acids, ketones, aldehydes, and triacyl glycerides, are potential biomarkers of gastric cancer^10^. Expression of aldehydes and ketones, which are metabolic products of β-oxidation, is increased in tumor tissues from gastric cancer patients.^20,21^ The results of the present study agree with those of a previous study showing that increased β -oxidation increases aldehydes production by gastric cancer cells^12^.

As a therapeutic approach, it is easy to cut off the supply of free fatty acids using a fatty acid synthase inhibitor. However, it is more difficult to restrict the supply of lipids and fatty acids to cancer by blocking receptors because cancer cells use endocytosis^22^ and autophagy as alternative routes^23^. Here, we show that fatty acids are the major source of β -oxidation, which is achieved through fatty aldehydes by lipid peroxidation. That is probably why cancer cells maintain a relatively high level of ROS as a free energy source, even though cancer cells show poor tolerance to ROS^24^. This observation concurs with that of a previous report showing that products of lipid metabolism, including fatty acids, ketones, and aldehydes, are biomarkers of oesophago-gastric cancer^10^.

Alternative supplies for ATP are glutamine^25^ and lactate^26^. However, metabolic flux analysis of glutamine revealed that it is catabolized to fatty acids through acetyl-CoA^27^. A recent study shows that glutamate, supplied by glutaminase 1, is associated with the malate aspartate shuttle that generates ATP from cytosolic NADH^6^. Therefore, glutamine is required for biosynthesis or energy metabolism in cancer. A recent report suggests that human NSCLC tumors use lactate in the TCA cycle^26^. Lactate uptake by cancer cells resulted in an increase in the amount of TCA cycle intermediates^26^. However, it is hard to find evidence that lactate supports major ATP production in cancer.

It is an undeniable fact that cancer cells produce ATP via mitochondrial oxidative phosphorylation; this is because the OCR of cancer cells is higher than that of normal cells^28^. Here, we found that about 40% of NADH produced by gastric cancer is dependent on ALDH3A1-mediated β-oxidation of fatty acids via conversion of fatty aldehydes to fatty acids. The remaining ATP production may depend on NADH production via β-oxidation. Recently, we reported that mitochondrial complex I is essential for drug resistance; indeed, production increases  i10-fold within 48 h of treatment with anti-cancer drugs^29^. Therefore, targeting ALDH3A1 and mitochondrial complex I using gossypol and phenformin results in almost complete depletion of ATP, which downregulates cancer cell growth through mTOR inhibition and further induces cell death by disturbing homeostasis. This type of energy metabolism is unique to cancer cells; normal cells use glucose to generate NADH via the TCA cycle. Therefore, targeting ALDH3A1 may have potential anti-cancer effects against gastric cancer.

## Supplementary information

SFigure 1-3

## Data Availability

All data generated or analyzed during this study are included in this published article (and its Supplementary Information file).
