# Supplementary material for: Gastric cancer depends on aldehyde dehydrogenase 3A1 for fatty acid oxidation
Source: Sci Rep. 2019 Nov 8;9:16313. doi: 10.1038/s41598-019-52814-1 (PMC6841934; doi:10.1038/s41598-019-52814-1)
Supplement: Supplementary file 1 — SFigure 1-3 [file 41598_2019_52814_MOESM1_ESM.pdf]

Supplementary information

Gastric cancer depends on aldehyde dehydrogenase 3 A1 for fatty acid oxidation

Jae-Seon Lee[1], Seung Hwa Kim[2], Soohyun Lee[1], Joon Hee Kang[1], Seon-Hyeong Lee[1],

Jae-Ho Cheong[2], and Soo-Youl Kim[1*]

Table of Contents

Supplementary materials and methods
Supplementary figures SF1-3
Supplementary table S1

Supplementary Materials and Methods

Cell culture

All gastric cancer cells were grown in RPMI 1640 medium (SH30027.01, HyClone, Logan, UT, U.S.A.) containing 10% fetal bovine serum (FBS) (SH30070.03HI, HyClone, Logan, UT, U.S.A.) and Cellmaxin (C3314-020, GenDEPOT). Cells were incubated at 37°C and maintained under 5% CO_2. Cells were transfected for 48 h with siRNA duplexes targeting human ALDH3A1 using Lipofector-Q Reagent (AB-LF-Q001, AptaBio, Yongin, Korea) and Plusfector Reagent (AB-PF-0001, AptaBio, Yongin, Korea), according to the manufacturer's instructions. Negative control cells were incubated with Lipofector-Q Reagent or Plusfector Reagent plus a negative siRNA (sc-37007, sc-44230) (Santa Cruz, Dallas, TX, U.S.A.). The ALDH3A1 siRNA sequences are shown in the Table.

**Gene name**	**Sequence**	
	**Sense（5'-3'）**	**Antisense（5'-3'）**
ALDH3A1 #1	GGACUAUGGAAGAAUCAUUdTdT	AAUGAUUCUUCCAUAGUCCdTdT
ALDH3A1 #2	GGUGAUUAAGAAGAUGAUUdTdT	AAUCAUCUUCUUAAUCACCdTdT

XF Cell Mito Stress analysis

Cells in 60 mm dishes were transfected with NT siRNA or ALDH3A1 siRNA (40 nM). After 24 h, transfected cells were seeded in XF cell culture microplates at a density of 30,000/well and incubated for 48 h at 37°C. To measure the oxygen consumption rate (OCR), cells were incubated in XF base medium supplemented with 10 mM glucose, 1 mM sodium pyruvate, and 2 mM L-glutamine, and then equilibrated in a non-CO2 incubator for 1 h before starting the assay. Samples were mixed (3 min) and measured (3 min) in an XFe96 extracellular flux analyzer (Seahorse Bioscience, North Billerica, Billerica, MA, U.S.A.). Oligomycin (1 µM), carbonilcyanide p-triflouromethoxyphenylhydrazone (FCCP, 1 µM), and rotenone/antimycin A (0.5 µM) were injected at the indicated times. Finally, the OCR was normalized in a SRB assay.

Relative quantitation of metabolites using liquid chromatography-tandem mass spectrometr y (LC-MS/MS)

Metabolites involved in energy metabolism were analyzed as described previously[11] using LC-MS/MS equipped with 1290 HPLC (Agilent, Santa Clara, CA, USA), Qtrap 5500 (ABSciex, Concord, Ontario, Canada), and reverse phase (Synergi fusion RP 50 × 2 mm) columns.

Relative quantitation of free fatty acids using gas chromatography-mass spectrometry (GC-MS)

Sample preparation

One million cells were harvested in 1 mL cold methanol after sequential washing with PBS. Next, cells were lysed by vigorous vortexing, acidified with HCl (final concentration, 25 mM), and then mixed with 50 μL internal standard (myristic acid-d27; 0.1 mg/mL). The sample was centrifuged at 13000 rpm for 10 min and the supernatant was collected in a fresh tube. Next, 3 mL iso-octane was added and the tube was centrifuged at 4000 rpm for 20 min. Finally, the upper layer was collected and dried under vacuum.

Fatty acid methyl ester (FAME) derivatization

The dried sample was reacted with 200 μL BCl3-MeOH (12% w/w; Sigma-Aldrich) at 60°C for 30 min. Next, 100 μL H2O and 100 μL hexane were added sequentially and mixed vigorously. The upper phase was collected after resting for 5 min. Then, 20–30 mg anhydrous sodium sulfate was added prior to GC/MS analysis. FAMEs (Sigma-Aldrich) were used to generate calibration curves without derivatization.

GC-MS

FAMEs were analyzed in a GC-MS system (Agilent7890A/5975C) fitted with a capillary column (HP-5MS; 30 m × 0.25 mm × 0.2 μm). Electron impact ionization was used in positive ion mode, with an injection volume of 1 μL and a split mode ratio of 10:1. Total analysis time was 73.7 min and the temperature gradient was as follows: hold at 50°C for 2 min; 50°C to 120 °C at 10°C /min; 120°C to 250°C at 3°C /min; hold at 250°C for 15 min; 250°C to 300°C at 35°C /min; hold at 300 for 5 min. The calibration range was 0.001–10 mg/mL (r2≥0.99). Data analysis was performed using MSD Chemstation software (Agilent E02.02.1431).

Relative quantitation of fatty acyl CoA using LC-MS/MS

Sample preparation

One million cells were harvested using 1.4 mL of cold methanol/H2O (80/20, v/v) after sequential washing with PBS and H2O. Cells were lysed by vigorous vortexing prior to addition of 100 μL internal standard (Malonyl-13C3 CoA; 5 μM). Chloroform was added and metabolites were extracted from the aqueous phase by liquid–liquid extraction. The aqueous phase was dried in a vacuum centrifuge and the sample was reconstituted with 50 μL of H2O/MeOH (50/50 v/v) prior to LC-MS/MS analysis.

LC-MS/MS

Fatty acyl CoA was analyzed by LC-MS/MS equipped with 1290 HPLC (Agilent), Qtrap 5500 (ABSciex), and reverse phase (Zorbax 300Extend-C18 2.1 × 150 mm) columns. Next, 3 μL sample was injected into the LC-MS/MS system and ionized by a turbo spray ionization source. Acetonitrile/H2O (10/90) with 15 mM ammonium hydroxide and acetonitrile with 15 mM ammonium hydroxide were used as mobile phase A and B, respectively. The separation gradient was as follows: hold at 0% B for 3 min; 0% to 50% B for 2 min; 50% to 70% B for 5 min; 70% to 0% B for 0.1 min; hold at 0% B for 4.9 min. LC flow was 200 μL/min and the column temperature was kept at 25°C. Multiple reaction monitoring was used in positive ion mode and the extracted ion chromatogram corresponding to the specific transition for each fatty acyl CoA was used for quantitation. The calibration range for fatty acyl CoAs was 0.1–10000 nM (r2≥0.99). Data analysis was performed using Analyst 1.5.2 software.

Cell cycle analysis

Cells were incubated with or without ALDH3A1 siRNA for 48 h, or with gossypol (5 μM) and/or phenformin (100 μM) for 24 h. Cells were then collected, washed twice with PBS, centrifuged at 1500 rpm for 5 min, and fixed overnight at 4°C with 70% ethanol. They were then centrifuged at 2000 rpm for 10 min, stained with PI + RNase solution for 30 min in the dark, washed with cold PBS, and then analyzed in a FACSCalibur flow cytometer (BD Falcon, Bedford, MA, USA).

TUNEL assay: cell death detection

A fluorometric TUNEL detection kit was used according to the manufacturer's instructions (11684795910; Roche Applied Science, Indianapolis, IN). Briefly, cells were treated with 5 μM gossypol and/or 100 μM phenformin (single and combination treatment) for 24 h, fixed for 10 min with 4% paraformaldehyde in PBS, permeabilized at 4°C for 2 min with 0.5% Triton X-100 in PBS, and incubated at 37°C for 1 h in the dark with the provided fluorescein-conjugated TUNEL reaction mixture in a humidified chamber. Omission of the TdT enzyme from the TUNEL reaction mixture was included as a negative control. The cells were then mounted with 4',6-diamidino-2-phenylindole (DAPI) mounting medium to visualize nuclei (Vectashield mounting medium; Vector Laboratories, Burlingame, CA). TUNEL- and DAPI-stained nuclei staining were examined under a Zeiss LSM780 confocal microscope (Carl Zeiss).

Supplementary Figure 1

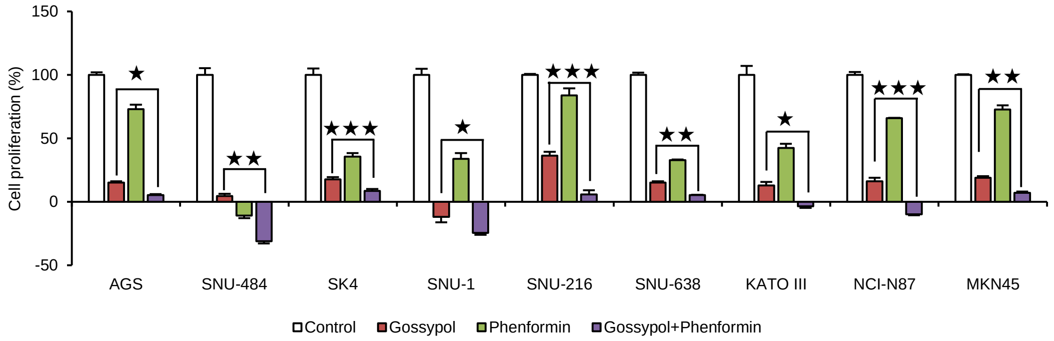

Fig. S1. Combined treatment with 5 μM gossypol and 100 μM phenformin for 48 h resulted in synergistic inhibition of gastric cancer cell growth, as determined in a SRB assay. Data are expressed as the mean and standard deviation of three independent experiments. *$p < 0.05$, **$p < 0.01$, ***$p < 0.001$.

Supplementary Figure 2

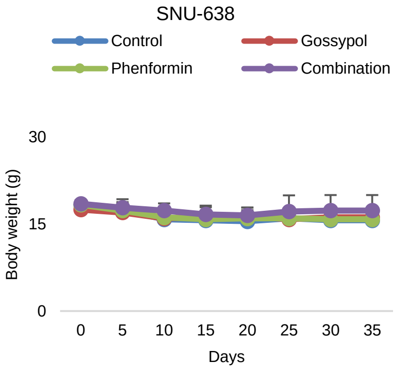

Fig. S2. Combined treatment with gossypol and phenformin results in synergistic suppression of tumors in a human gastric cancer xenograft mouse model. Body weight of four treatment groups was measured weekly after inoculation of SNU-638.

Supplementary Figure 3

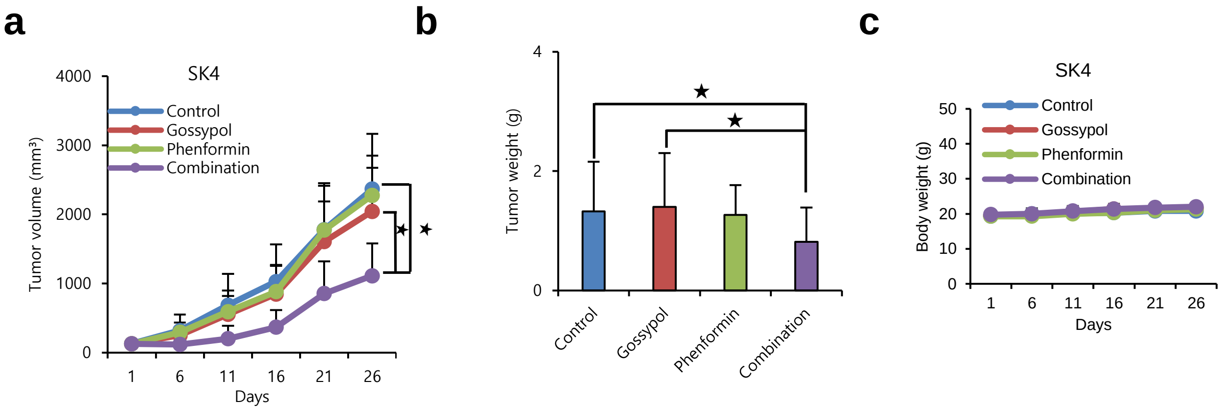

Fig. S3. Combined treatment with gossypol and phenformin leads to synergistic suppression of tumor growth in a human gastric cancer xenograft mouse model.

(a) SK4 (5×10^6) cells were injected into 6–8-week-old BALB/c nude mice. When the volume of the tumor mass reached 130 mm^3, mice were assigned randomly to one of four treatment groups: vehicle control, gossypol, phenformin, and gossypol plus phenformin (n=6 per group). Gossypol (80 mg/kg body weight), phenformin (100 mg/kg body weight), and vehicle were administered orally 6 days/week. The graph shows a synergistic reduction in tumor growth after combined treatment with gossypol and phenformin (measured using calipers). (b) Final weight of subcutaneous tumors derived from SK4 cells. (c) Body weight of mice was measured weekly after inoculation of SK4. *$p < 0.05$, **$p < 0.01$, ***$p < 0.001$.

	Overall	ALDH3A1-negative (n=459, 40.5%)	ALDH3A1-positive (n=673, 59.5%)	p-value
Age (y)	56.67 ± 12.17	56.83 ± 12.11	56.56 ± 12.21	0.707
Sex				0.116
Male	739 (65.3)	312 (68.0)	427 (63.4)	
Female	393 (34.7)	147 (32.0)	246 (36.6)	
Histology				0.059
Differentiated	366 (32.3)	163 (35.5)	203 (30.2)	
Undifferentiated	766 (67.7)	296 (64.5)	470 (69.8)	
Lauren Classification				0.41
Intestinal	540 (49.0)	222 (51.5)	318 (47.4)	
Diffuse	524 (47.5)	195 (45.2)	329 (49.0)	
Mixed	38 (3.4)	14 (3.2)	24 (3.6)	
Size (mm)	56.11 ± 29.74	56.23 ± 30.18	56.03 ± 29.46	0.911
pT stage				0.001
pT2	228 (20.2)	116 (25.3)	112 (16.6)	
pT3	160 (14.1)	71 (15.5)	89 (13.2)	
pT4a/b	744 (65.7)	272 (59.2)	472 (70.2)	
pN stage				0.051
pN0	338 (29.9)	149 (32.5)	189 (28.1)	
pN1	198 (17.5)	84 (18.3)	114 (16.9)	
pN2	219 (19.3)	94 (20.5)	125 (18.6)	
pN3a/b	377 (33.3)	132 (28.7)	245 (36.4)	
TNM stage				0.003
Stage I	145 (12.8)	76 (16.6)	69 (10.3)	
Stage II	293 (25.9)	120 (26.1)	173 (25.7)	
Stage III	604 (53.4)	237 (51.6)	367 (54.5)	
Stage IV	90 (8.0)	26 (5.7)	64 (9.5)	

Table. S1.

Full length gels for Figure 1c

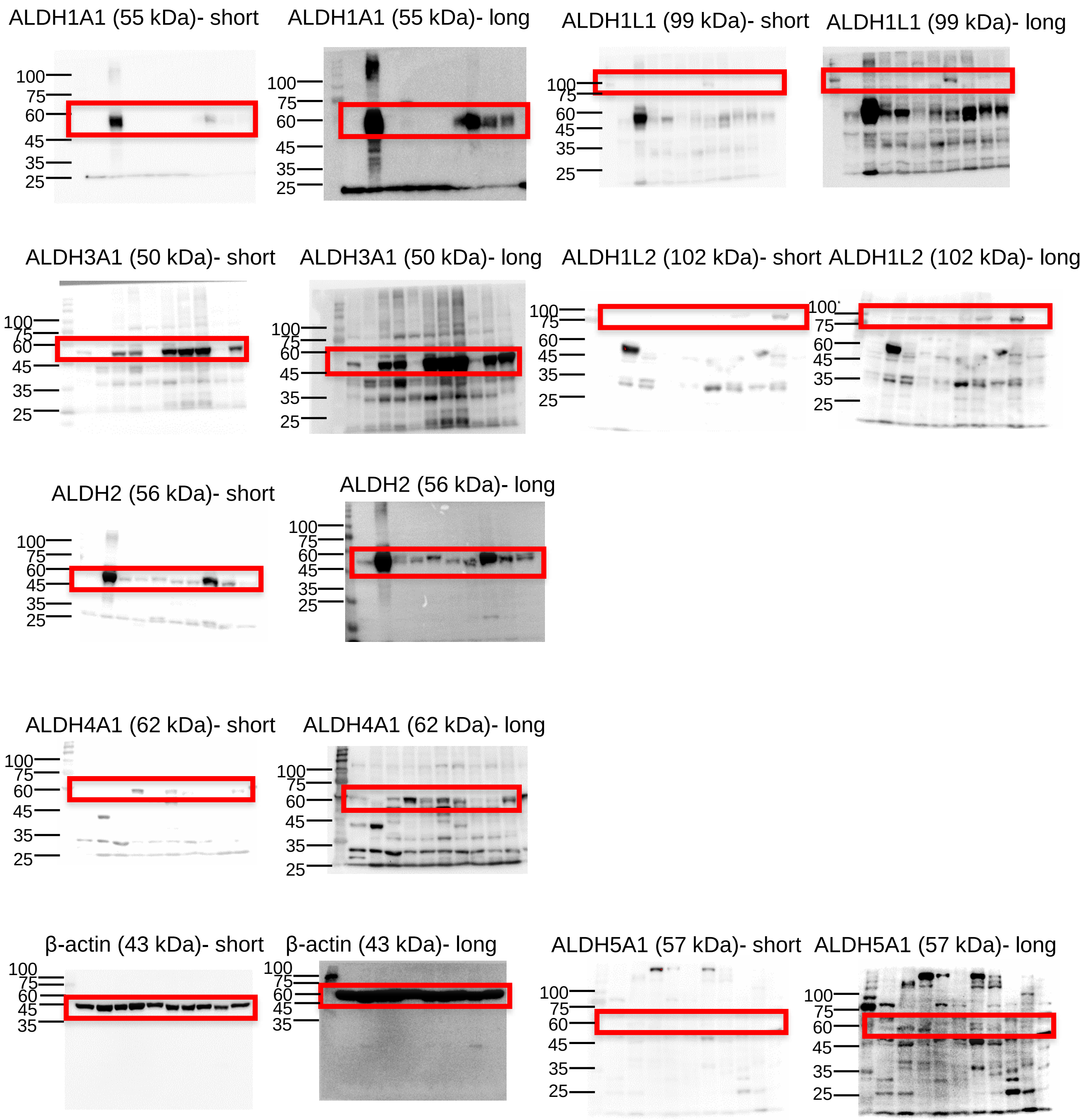

Areas of the gel shown in Figure 1b are marked in red. Predicted molecular weights for ALDH isoforms (short and long exposure) and β-actin are as indicated.

Full length gels for Figure 1d

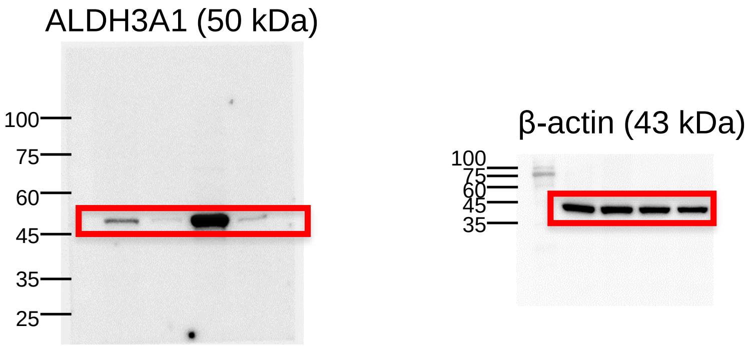

Areas of the gel shown in Figure 1d are marked in red. Predicted molecular weights for ALDH 3A1 and β-actin are as indicated.

Full length gels for Figure 5d

Cyclin D1 (36 kDa) - short exposure

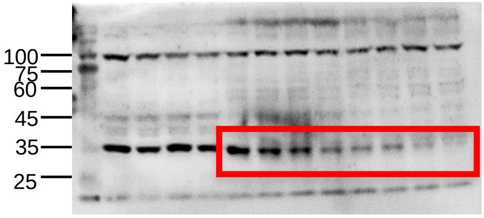

Cyclin D1 (36 kDa) – long exposure

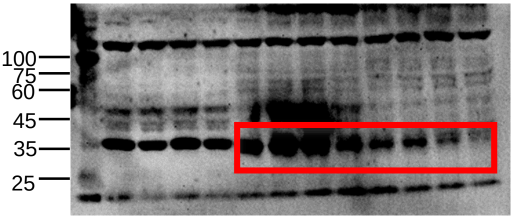

β-actin (43 kDa)

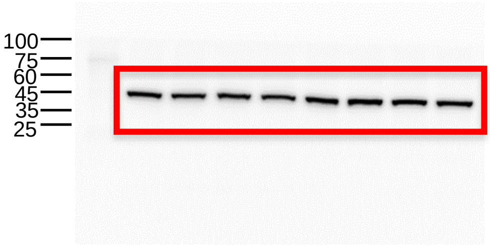

Areas of the gel shown in Figure 5d are marked in red. Predicted molecular weights for Cyclin D1(short and long exposure) and β-actin are as indicated.
